# Overview of thermal ablation devices for treating precancerous cervical lesions in low-resource settings

**DOI:** 10.7189/jogh.12.03089

**Published:** 2022-12-29

**Authors:** Taylor Boles, Mila Pontremoli Salcedo, Cesaltina Lorenzoni, Nafissa Osman, Ellen Baker, Kathleen Schmeler, Jennifer Carns

**Affiliations:** 1 Rice360 Institute for Global Health Technologies, Rice University, Houston, Texas, USA; 2 Department of Gynecologic Oncology and Reproductive Medicine, The University of Texas MD Anderson Cancer Center, Houston, Texas, USA; 3 Ministry of Health, Maputo, Republic of Mozambique; 4 Universidade Eduardo Mondlane (UEM), Maputo, Mozambique; 5 Department of Bioengineering, Rice University, Houston, Texas, USA

In 2020, there were an estimated 604 000 cases and 341 000 deaths from cervical cancer [1], with 91% of these deaths occurring in low- and middle-income countries (LMICs) [2]. While screening programs have led to a dramatic reduction in cervical cancer rates in high-income countries, their implementation in LMICs has faced many challenges [3]. Screen-and-treat programs, where screening is achieved with human papillomavirus (HPV) testing and/or visual inspection with acetic acid (VIA), have limited impact when feasible treatment options for precancerous cervical lesions are not available. Cryotherapy has historically been recommended as an ablative treatment, but implementation challenges related to obtaining and transporting the necessary refrigerant gas have limited its use in many low-resource settings [4,5]. Similar to cryotherapy, thermal ablation does not require anaesthesia and can be provided by a variety of health care providers [4]. Thermal ablation devices are simple to use, lightweight, portable, and easier to implement than cryotherapy in many settings, while the necessary treatment time for thermal ablation is much shorter than for cryotherapy [4,6]. When thermal ablation is used to treat precancerous cervical lesions at 100-120°C for 20-45 seconds, the depth of necrosis is sufficient, with cure rates comparable to those achieved with cryotherapy [6].

For women who screen positive by HPV testing and/or VIA, ablative therapy is appropriate if the squamocolumnar junction (SCJ) is entirely visible, the lesion occupies less than 75% of the cervix and can be completely treated with the ablation probe, the entire lesion can be visualized (ie, does not enter the endocervical canal), and it is not suspicious for cancer or glandular disease [4,6]. In many countries, it is estimated that 70%-80% of women who require treatment are eligible for ablative therapy [7]. Thermal ablation has become an attractive alternative to cryotherapy, is now recommended by the WHO [4], and has been recently incorporated into the national guidelines for many LMICs [8,9]. As many countries are exploring the transition from cryotherapy to thermal ablation, we provide an overview of three currently available thermal ablation devices and present practical considerations for implementing thermal ablation in low-resource settings.

## OVERVIEW OF THERMAL ABLATION DEVICES FOR LOW-RESOURCE SETTINGS

Specifications for commercially available thermal ablation devices were obtained from their user manuals and via email communication with manufacturers. Three point-of-care thermal ablation devices are summarized in **Table 1** – the Liger thermocoagulator, the WISAP cervical cold coagulator, and the MedGyn thermal ablation system. All three devices are CE marked and available in low-resource settings.

**Table 1:** Summary of point-of-care thermal ablation devices

	Liger	MedGyn	Wisap
**Model No.**	HTU-110	MTA-100	C3
**Cost**	US$1500 (includes 4 probes)*	US$1550 (includes one tip)*	€2326 ( ~ US$2900) (includes 2 probes and 2 sliders)*
**Cost of additional probes/tips**	US$100 each*	$245 USD each*	€602 each ( ~ US$725)*
**Power sources available**	Rechargeable battery	Rechargeable battery	Wall plug or rechargeable battery (power supply included)
**Number of batteries/chargers included**	2 batteries, 1 charger	2 batteries, 1 charger	1 battery, 1 charger
**Number of cycles per battery charge**	~ 30-60	~ 100 (depends on length of treatment cycle)	~ 140
**Low battery indicator**	Yes	Yes	Yes
**Probe treatment temperature**	100°C	100°C	100°C
**Probe heating time**	8 s	30-50 s	20-30 s
**Treatment time**	20-60 s (beeps after 1/4, 1/2, and 3/4 of treatment time)†	20, 40, or 60 s†	60 s (beeps after 30, 45 and 60 s)
**Built in timer**	Yes	Yes	Yes
**Light source**	LED illumination	LED illumination	LED illumination
**Temperature display**	No‡	Yes	No
**Probe options**	16mm flat, 19mm flat, 19mm nipple	19mm flat, 19mm x 5mm convex, 19mm x 10mm endocervical, 25mm x 7mm endocervical, 25mm flat	17 mm flat, 20 mm flat, 20 mm nipple
**Removable probes**	Yes	Only probe tip is removable, shaft is not removable	Yes
**Heat protection**	Yes, via an integrated plastic ring that covers the heating element.	Yes, via an integrated plastic ring that covers the heating element in the tip.	Yes, via a retractable sleeve that covers the heating element.
**Probe lifetime**	~ 100 cycles	at least 50 cycles	~ 500 cycles
**Method of disinfection**	Removable probe can be soaked in Cidex or autoclaved. Autoclaving may reduce lifetime of probe.	Hand unit and shaft can be cleaned with CaviWipes, Cavicide, and enzymatic detergent, followed by wiping with damp cloth. Care should be taken to avoid getting water inside the unit. The tips can be autoclaved.	Removable probe can be soaked in Cidex. Probes should not be autoclaved.

HTU-110 – Liger thermocoagulator, MTA-100 – Medgyn thermal ablation system, C3 – WISAP cervical cold coagulator

*Pricing should be verified with manufacturer; discounts may be available when buying in volume.

†Treatment time is programmable.

‡Temperature is internally monitored; error signal will be generated if temperature is out of range.

### Liger thermocoagulator

The Liger thermocoagulator (HTU-110) includes the control unit, four thermal probes, two battery packs, and a charging base [10]. The thermal probe heats to 100°C in eight seconds and remains at 100°C for the duration of the treatment time. The treatment time can be configured by the user to between 20 and 60 seconds, with a default of 30 seconds. The probe has an integrated plastic ring that prevents the side of the probe tip from inadvertently coming into contact with tissue while heated. A white light LED is integrated into the handle to aid visibility when placing the probe in contact with the cervix. The heating button allows the operator to activate and deactivate heating of the probe. A set of four blue LED’s blink as the probe heats to 100°C and light up as the treatment timer runs. Audible beeps are heard when 1/4, 1/2, and 3/4 of the treatment time has elapsed, as well as upon completion of treatment. The probe temperature is monitored internally and signals an error to the user if the temperature is out of range. Three probe options are offered for the device – flat probes that are 16mm and 19mm in diameter and a flat probe with a small nipple that is 19mm in diameter. Each probe is expected to last for about 100 treatment cycles before needing replacement.

High-level disinfection of the removable thermal probe and shaft is recommended. Liger provides a validated disinfection procedure that includes soaking the probe in Cidex® followed by repeatedly rinsing the probe with water. Probes may also be autoclaved, though the use of an autoclave may reduce the lifetime of the probe.

### MedGyn thermal ablation system

The Medgyn thermal ablation system (MTA-100) includes the control unit, thermal tip, and power supply [11]. The thermal tip heats to 100°C in 30-50 seconds and remains at 100°C for the duration of the treatment time. The tip has an integrated plastic ring that prevents its side from inadvertently coming into contact with tissue while heated. A ring of six white light LED’s is integrated into the handle to aid visibility when placing the tip in contact with the cervix. The heating button allows the operator to activate and deactivate heating of the tip. A LED and audible beep indicate that the device is heating. A screen displays the temperature of the tip during heating and treatment. Once heated, buttons on the device allow the user to start a treatment timer for 20, 40, or 60 seconds. Five tip options are available, including flat tips that are 19 and 25mm in diameter, endocervical tips that are 19 and 25mm in diameter, and convex tips that are 19mm in diameter. Each probe tip is expected to last for up to 50 treatment cycles before needing replacement.

High level disinfection of the removable thermal tip and shaft is recommended. MedGyn provides a disinfection procedure that consists of wiping the tip and shaft with a CaviWipe® cloth and spraying Cavicide® on the tip. The tips may also be autoclaved.

**Figure:**
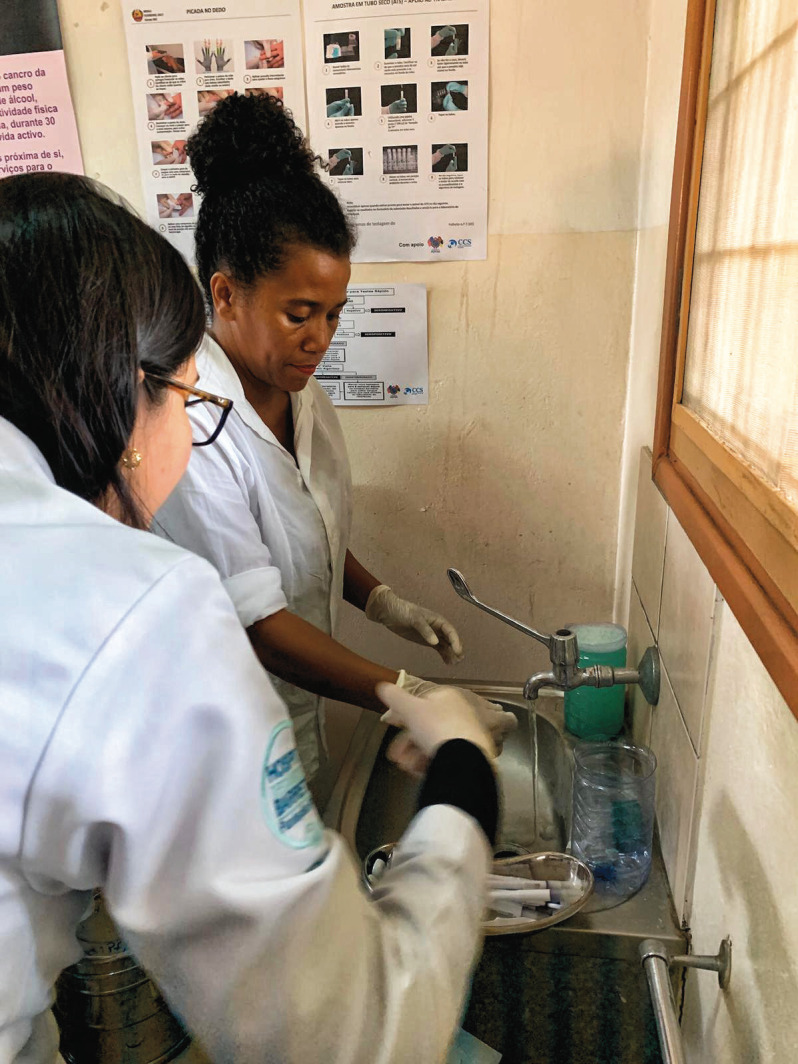
Photo: Cleaning and disinfection of thermal ablation probes in a clinic in Maputo, Mozambique. Source: Choi Lin Machiana and Viviane Andrade, used with permission.

### WISAP cervical cold coagulator

The WISAP cervical cold coagulator (C3 set) includes the control unit, thermal probe, slider, and power supply [12]. The thermal probe provides heat at 100°C for 60 seconds; the distal end of the probe is teflon-coated to prevent the adherence of any tissue to the probe. The included slider can be placed over the thermal probe to provide heat-protection for the vaginal walls when the probe is removed. A white light LED is integrated into the handle to aid visibility when placing the probe in contact with the cervix. The heating button allows the operator to activate and deactivate probe heating. A green LED blinks as the probe heats to 100°C, and stays constantly green once the treatment temperature is reached. The operator can activate the timer by pressing the timer button on the handle, and audible beeps can be heard after 30 seconds, 45 seconds, and 60 seconds of treatment. Probe options include flat probes that are 17mm and 20mm in diameter, as well as a 20mm endocervical probe with a nipple. According to the manufacturer, the probe should withstand ~ 500 use-disinfection cycles before needing to be replaced.

High level disinfection of the removable thermal probe and slider is recommended. WISAP provides a validated disinfection procedure that consists of washing with sterile water and a soft medical brush, submersion in CIDEZYME® Enzymatic Detergent Solution, rinsing with clean water and drying, submersion in Cidex OPA HLD solution, and rinsing with sterile water.

## CONSIDERATIONS FOR IMPLEMENTATION

To expand access to cervical cancer screening services, there have been efforts to integrate “screen and treat” programs with family planning or HIV/AIDS services, which are widely available, even in under-resourced health clinics. Although thermal ablation has many advantages over cryotherapy, some implementation challenges in these settings still need to be considered.

Costs considerations should include the basic cost of the handset and associated probes and batteries, and the cost of replacement probes. The probes/heating element of all three devices require replacement after a certain number of uses. If a US$200 probe is expected to last 100 heating cycles, the cost of using the device would be US$2 per treatment. This can be a significant expense and should be considered as a maintenance expense alongside the base equipment cost. Costs associated with disinfection should also be factored into the treatment cost.

Ease of use, power requirements (eg, batteries, time to charge and number of treatments per charge), treatment aids (eg, an integrated light source and timer), safety features (eg, shielding of the heated probe to avoid burns) and cleaning and disinfection requirements should be considered in selecting a device. Availability of an autoclave or disinfection solutions, as well as availability of running water can impact the ability to safely use these devices.

Devices that are intuitive, simple to use, easy to assemble, operate and clean, with few controls and well-integrated treatment aids (timers, light source) will require less training, particularly for those experienced in providing cryotherapy treatment.

The durability of, and logistics associated with replacing the handsets, batteries, and probes should also be considered. Surge protection while charging the batteries may extend the battery life. Availability of replacements for malfunctioning components (eg, probes/batteries) and the associated lead time and shipping costs for these parts may also be a significant factor to consider.

## CONCLUSION

Thermal ablation is an effective and affordable alternative to cryotherapy for the treatment of precancerous cervical lesions. It is particularly well-suited for use in low-resource settings because thermal ablation devices are lightweight, portable, and simple to implement and use, while requiring a much shorter treatment time than cryotherapy. These thermal ablation devices provide an opportunity to expand cervical cancer prevention efforts in low-resource settings.
